# Liquid-crystalline nanoarchitectures for tissue engineering

**DOI:** 10.3762/bjnano.9.22

**Published:** 2018-01-18

**Authors:** Baeckkyoung Sung, Min-Ho Kim

**Affiliations:** 1 Liquid Crystal Institute and Chemical Physics Interdisciplinary Program, Kent State University, Kent, OH 44242, USA; 2 Department of Biological Sciences, Kent State University, Kent, OH 44242, USA

**Keywords:** biocolloid, biopolymer, cell-matrix interaction, mesophase, regenerative medicine

## Abstract

Hierarchical orders are found throughout all levels of biosystems, from simple biopolymers, subcellular organelles, single cells, and macroscopic tissues to bulky organs. Especially, biological tissues and cells have long been known to exhibit liquid crystal (LC) orders or their structural analogues. Inspired by those native architectures, there has recently been increased interest in research for engineering nanobiomaterials by incorporating LC templates and scaffolds. In this review, we introduce and correlate diverse LC nanoarchitectures with their biological functionalities, in the context of tissue engineering applications. In particular, the tissue-mimicking LC materials with different LC phases and the regenerative potential of hard and soft tissues are summarized. In addition, the multifaceted aspects of LC architectures for developing tissue-engineered products are envisaged. Lastly, a perspective on the opportunities and challenges for applying LC nanoarchitectures in tissue engineering fields is discussed.

## Review

### Introduction

Liquid crystals (LCs) are ubiquitous in our life [1]. On one hand, LC materials play a central role in modern technology and industry ranging from electronic display devices to optical communication networks [2–4], thanks to their softness and flexibility, rapid molecular self-organization/reorganization, and sensitive responsivity to external stimuli [5]. For this reason, many researchers have been striving for decades to invent biocompatible LC nanostructures for biomedical applications [6–7]. These works cover medical imaging and spectroscopy instruments [8–9], diagnostic biosensors [10], microlens devices [11], soft actuators [12–13], and drug delivery systems [14–15]. On the other hand, LCs are core materials in the living body [16]. For example, cell membranes [17] and chromosomes [18–19] exhibit LC-like phases, and some pathological states are closely related to LC formation processes, such as those of amyloid fibrils [20]. Consequently, complex self-organization dynamics of living systems have been modeled and analyzed as LC structures.

LCs can be not only versatile model systems for understanding self-organization mechanisms in biological phenomena, but also robust engineering platforms to create novel functional nanomaterials and microdevices for tissue repair. Tissue mimicry is a strongly demanded feature in every kind of templates and scaffolds for tissue regeneration [21–22]. A biologically derived or biocompatible synthetic LC material can provide close resemblance to those in native tissues, by forming self-assembled 3D nanoarchitectures. Nevertheless, this aspect has not yet been sufficiently recognized among scientists in the field of tissue engineering and regenerative medicine, or even among experts in fields of LC technology.

The objective of this review article is to deal with this issue with a multidisciplinary point of view. First, the ultrastructures and phase behaviors of LCs found in biological tissues in vivo as well as biocolloid systems in vitro are introduced, with a specific emphasis on the nanoscale geometries of elementary LC phases. Then, recent research efforts for applying LC nanoarchitectures to tissue engineering are summarized and discussed, in the context of their applications to cell templates and scaffolds for regenerative medicine. In particular, it is discussed how varying nanoarchitectures in different LC orders have been realized in several tissue engineering topics. Lastly, a perspective on the opportunities and challenges for applying LC nanoarchitectures to tissue regeneration purposes is discussed.

### Liquid crystalline phases of biological polymers and colloids in vitro

#### Geometry in biological LCs

**Figure 1:**
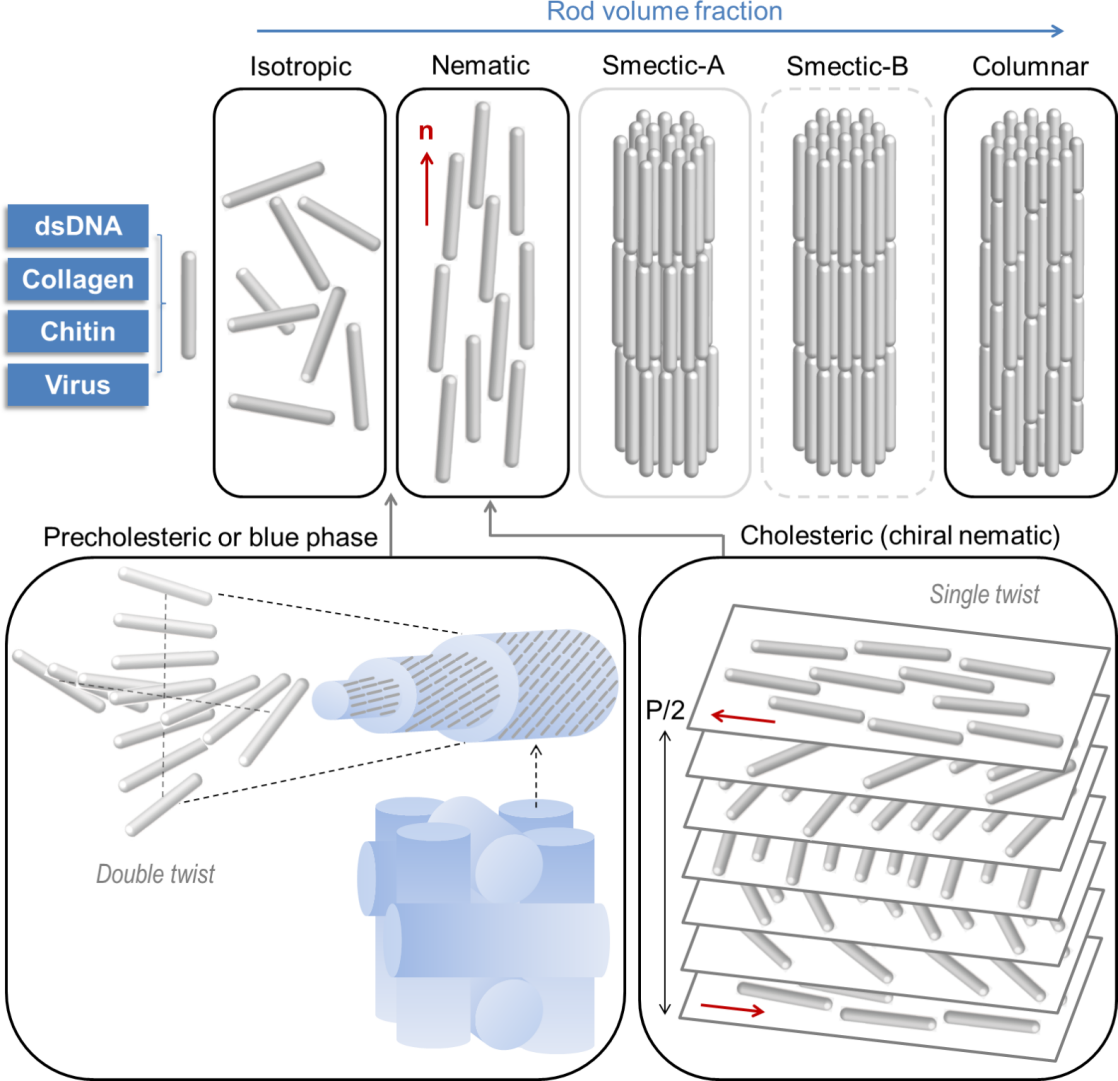
Lyotropic liquid-crystalline phase transition of a dispersion of rod-like particles as a function of rod volume fraction (i.e., rod concentration). A biopolymer (such as dsDNA, collagen, chitin and cellulose) or a filamentous virus (such as fd, M13 and TMV) is represented by a single hard rod. The phase behavior and the path of transitions vary for different types of rods and physicochemical conditions. The mean orientation of rods in nematic phase, called director, is denoted as **n**. The director undergoes a twist along a single axis (cholesteric phase) or along two axes (precholesteric or blue phase). In the cholesteric phase, the distance for rotating **n** by 360° is defined as the helical pitch (P). In the blue phase, a body-centered cubic cell (blue phase I) or a simple cubic cell (blue phase II; depicted in Figure 1) can be formed based on the double-twist cylinders as building blocks. In the smectic phase, the translational symmetry along **n** is broken without and with positional order, for smectic-A and smectic-B, respectively. This lamellar structure is absent in the columnar phase and positional order dominates the system with unidirectionality. Note that, in DNA dispersions, the smectic-A phase appears only under special conditions and the smectic-B phase has not been found.

Lyotropic LC phases are observed in the aqueous dispersions of various types of biological rod-like building blocks, such as DNA, collagen, chitin, viruses, as well as phospholipids and cellulose [23–24]. In test tubes, biocolloidal systems can be finely tuned to form self-organized structures by complex interplays between entropic and enthalpic driving forces, electrostatic and viscoelastic effects, and hydrophilic and hydrophobic molecular interactions [25–27]. Even simple solutions of purified biopolymers and biocolloids, considered as rod-like particles, may exhibit multiple phases of self-organization depending on the rod concentration, ionic conditions, confinements, and shear forces [28–29]. Figure 1 shows an example of LC phases of biocolloids, especially focused on the B-form of double-stranded DNA (dsDNA). Here the rod-like fragments (50 nm in length) of dsDNA undergo a phase transition in dependence on the rod concentration, dissolved in the 0.25 M ammonium acetate buffer. The polymorphic LC phases and their complex geometries of DNA molecules have been thoroughly explored by Livolant and colleagues [30–31]. As the DNA concentration increases, the mesophase of DNA molecules transits from the isotropic phase, blue phase or precholesteric phase (below 160 mg/mL), cholesteric phase (160–380 mg/mL), and columnar hexagonal phase (380–670 mg/mL) to the crystalline phase. The isotropic phase is the solution at a low rod concentration and lacks orientational and positional orders. In the nematic phase, orientational order is introduced by volume exclusion effects, in a way that the rods are aligned more or less parallel to one another. The mean direction of this long-range orientational order is called the director. If the constituent rods have intrinsic chirality, the cholesteric phase (also termed as chiral nematic phase) is induced by continuously twisting the nematic alignment, which can be manifested as the fingerprint texture between crossed polarizers. This texture originates from the helical structure of the director field in the cholesteric phase, where the distance required to rotate the director by an angle of 2π corresponds to the cholesteric pitch. In cross-sectional electron microscopy images, the cholesteric organization (with single twist) was shown to be displayed as a regular series of arced patterns, which is referred to as Bouligand structure [32–33] and described as a twisted plywood model. Between the isotropic and the cholesteric phase, the blue or precholesteric phase may exist, which is a double-twist configuration of rods ordered in a cylindrical geometry. The blue phase consists of double-twist cylinders that self-organize into a body-centered cubic cell (blue phase I) or a simple cubic cell (blue phase II). In some special systems, it has been shown that the smectic-A phase can be obtained from concentrated DNA solutions [34]. In the smectic-A phase, translational symmetry is broken along the director of the nematic phase, so that the rods that form a multilayer organization are oriented perpendicular or oblique to the layers, and are in a liquid-like order within each layer. In smectic-B, this liquid-like order is replaced by a hexagonal positional order. The smectic-B phase has not been observed in DNA systems. In the columnar hexagonal phase, the rods are unidirectionally aligned by maintaining a local hexagonal order. The lamellar order is no longer preferred in this phase. The columnar phase is fundamentally different from the crystalline phase in the sense that the rod suspensions exhibit fluidity and viscoelasticity. The crystalline phase is a complete solid state where the rods are fully ordered and tightly bound to each other in a 3D hexagonal phase or in a 3D orthorhombic phase.

#### Examples of bioengineered materials displaying LC phases

DNA is one of the most frequently used biomolecules in the field of biomedical engineering and bioinspired technology [35]. The DNA mesophase has been exploited for several applications, including biosensors [36] and drug delivery systems [37]. For tissue regeneration, the mostly studied biomaterials are collagen and chitin, which are, respectively, protein-based and glucose-based biopolymers [38–39]. When denatured, collagen and chitin can be transformed into gelatin and chitosan, respectively, which are also widely-used raw materials in biomedicine [40–41]. All of these macromolecular chains can be considered as semiflexible rod-like particles that exhibit intrinsic chirality [23]. Consequently, the cholesteric phase is the most commonly found LC organization in living matter [42]. Collagen type I, the major fibrous protein component in the extracellular matrix (ECM), has been shown to exhibit an isotropic-to-cholesteric phase transition (via the precholesteric phase) as the collagen concentration increases in acidic environments [43–44]. For example, solutions of type-I collagen triple helices, at collagen concentrations above 100 mg/mL in 0.5 mM acetic acid, exhibit strong birefringence with a fingerprint texture in polarizing microscopy. In the coexistence regime of isotropic and nematic phases of collagen, spindle-shaped cholesteric tactoids appear in the isotropic medium. The cholesteric droplets of concentrated suspensions of α-chitin and chitosan have been reported [45], and nematic order was also confirmed in the solutions of collagen type IV [46]. Similarly, the cholesteric phase behavior of cellulose fibrils is well established. For all these cases, the isotropic–nematic transitions can be quantitatively characterized by Onsager’s theory [44]. In addition, various types of rod-like viruses (fd, M13, f1 virus and TMV) have been considered as template materials for tissue regeneration. The rod-like viruses have been shown to undergo a series of lyotropic phase transitions from isotropic, cholesteric, smectic, and columnar, to crystalline phases, as the rod volume fraction increases [47–48].

### Liquid crystalline architectures in tissues and cells in vivo

**Figure 2:**
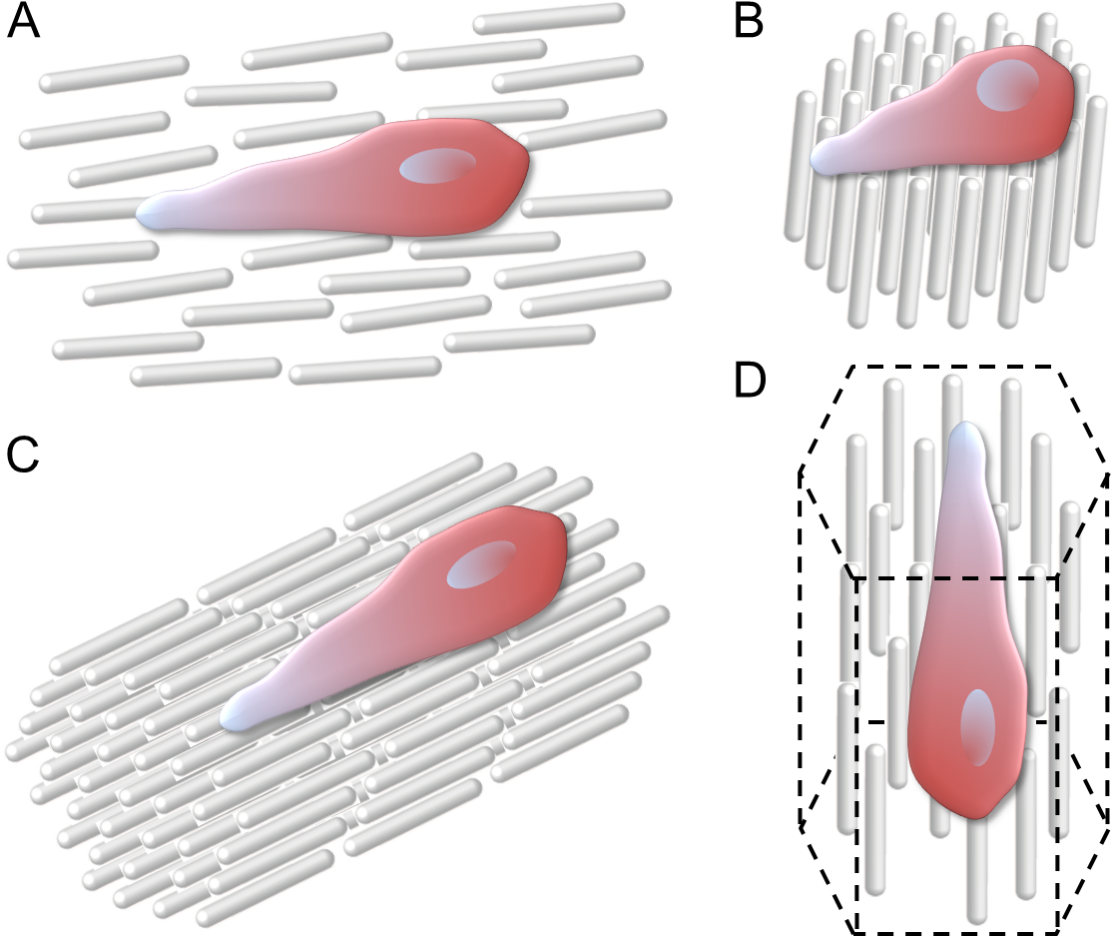
Architectures of liquid crystalline scaffolds and their interactions with adherent cells. Each rod represents a constituent polymer, colloid or molecule. (A) A cell attached on a substrate in nematic order; (B) a cell attached on a smectic substrate (perpendicular or oblique to the rod alignment); (C) a cell attached on a surface of smectic scaffold (parallel to the rod alignment); (D) a cell entrapped in a columnar scaffold. Note that the cells are elongated along the direction of rod alignment in panels A, C, and D.

Living systems are based on highly crowded milieus to drive the formation of dense packings of the constituent molecules [49]. This molecular crowding effect is a well-known driving force that directs the LC self-organization of biomacromolecules in vivo. As a consequence, a variety of ordered architectures in living tissues resemble analogues of LC phases, related to diverse biological functions [50–52]. Figure 2 shows a schematic of LC nanoarchitectures interacting with cells.

The LC order of the biopolymers has three major implications for the biological systems: (1) cell–matrix interaction, (2) formation of ECM-based connective tissues, and (3) cellular and subcellular level physiology. First, the spatially anisotropic alignment of ECM fibers is known to control the cell shape and motion [53]. During development, growth, and proliferation, the 2D/3D topography of ECM affects the cell spreading, focal adhesion strength, and intercellular mechanical signaling [54]. For example, the guided migration of cells in the ECM was shown to result in enhanced cellular reorganization and facilitated tissue morphogenesis [55]. Mechanical stiffness of tissues is an important physical cue for modulating the behavior of adhered or embedded cells [56–57]. The LC architectures of ECM fibers may influence the functionality of target tissue by altering the elastic modulus of tissue [58].

#### Hard tissues

Skeletal tissues are mineralized compact matrices of ordered biopolymers [59]. In vertebrates, bones consist of a dense mesophase of collagen fibrils [60], which has been believed to render mechanical stiffness to the skeletal tissues [61–62]. The fundamental unit of compact bones, called osteon, is a cylindrical structure which is composed of concentric lamellae. In each coaxial lamellar matrix, collagen fibrils are aligned parallel to each other, helically running around a central canal [63]. This geometry corresponds to the twisted plywood structure with a curvature [64]. Similar architectures can be found in the exoskeletal tissues of invertebrate animals, which are mineralized dense matrices of aligned chitin nanofibrils [16]. In these tissues, ultrastructures analogous to cholesteric mesophase are displayed as Bouligand structures [32].

#### Soft connective tissues

Mesophase organizations of the ECM fibers can play important roles not only in the optimization of mechanical properties of the tissue itself, but also in the regulations of functions of embedded cells [65]. The assembly structure of oriented collagen fibers in consecutive layers with a finite twist angle (a variation of the twisted plywood structure) is the most common arrangement found in hydrated soft connective tissues in vertebrates [66] and bears a strong resemblance to the cholesteric order. For example, in the annulus fibrosus disci intervertebralis, a cartilaginous endplate component of intervertebral discs, the inter-twisting lamellae of oriented collagen matrices and their translamellar cross-bridge networks is postulated, which affect the morphology of interlamellar chondrocytes in association with disc aging and degeneration [67]. Moreover, local changes in the plywood structure of corneal collagen fibrils may affect the biomechanical behavior of the cornea, which is in close relation to the physical maintenance of intraocular pressure [68].

#### Skin, nerve, and muscle cells and subcellular structures

Cell membranes are well-defined LC lamellae [69]. The epithelial and nerve cells exhibit mesophase organizations, as evidenced by nematic-like cytodynamics in epithelial cell monolayers [70] and smectic-like ultrastructures in the myelin sheath [71]. Muscle cells and tissues can be in nematic order [72–73]. Cholesteric orders of dsDNA have been observed in the packed dsDNA molecules in sperm heads [74–75] and in the dinoflagellate and bacterial chromosomes [76–78].

### Tissue-mimectic liquid-crystalline biomaterials

**Figure 3:**
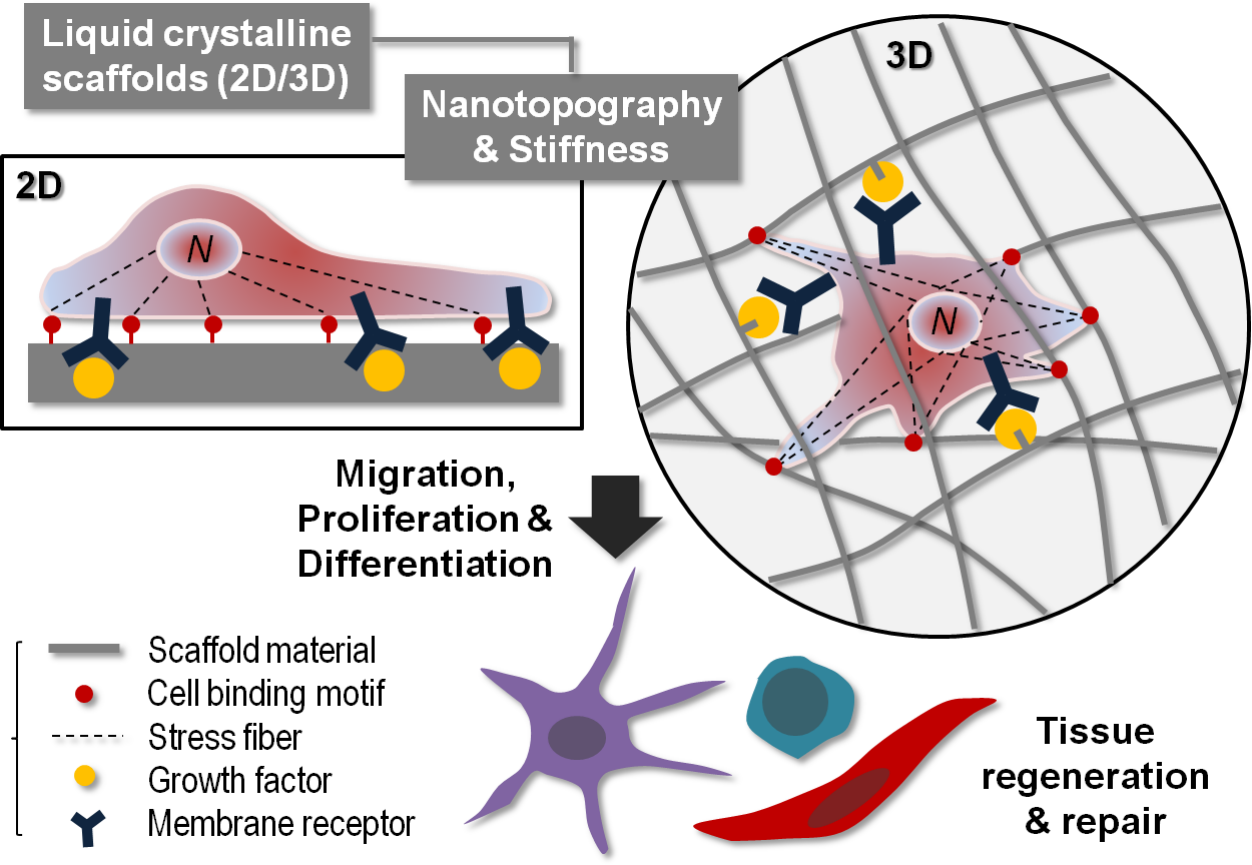
Physicochemical cues in tissue engineering scaffolds for controlling cellular responses. Cells may be attached on the 2D surface or embedded in the 3D structure. The cell behavior is governed by the physical factors (2D/3D topography and mechanical stiffness) and the biochemical factors (cell binding and molecular stimulation). The mechanical feedback from the scaffold is transmitted to the cell nucleus (N) via actin bundles (stress fibers). The chemical signal is generated by growth factors and then transduced by transmembrane receptors. By regulating physicochemical cues in the scaffold, the cell migration, proliferation, and differentiation can be controlled suitable for clinical uses.

3D self-organization and self-assembly of biocompatible LC structures satisfy essential characteristics required for tissue engineering materials and devices, coupled with microscale and nanoscale biotechnologies [79–80]. Complex formations of biomimectic patterns [81], based on the LC organization, can be utilized for engineering soft connective tissues [66], mineralized and calcifying hard tissues [82–83], and tissue-resembling composites and materials [84]. As shown in Table 1, the potential clinical applications of LC biomaterials include the regeneration of (1) acellular tissue such as bones, teeth (dentine and enamel), and cornea, (2) cell-ECM complexes such as spinal cords, tendons, and skin layers, and (3) cellular tissue such as blood vessels and peripheral nerves. Figure 3 summarizes the biologically-relevant physicochemical interactions occurring between scaffolds and cells.

**Table 1:** Summary of examples on liquid crystalline templates and scaffolds for tissue engineering applications.

material	structure	function	application	reference

purified collagen type I	cholesteric gel substrate	osteogenesis of human mesenchymal stem cells via aligned 2D growth	bone regeneration	[86]
		formation of human corneal epithelium with optical transparency	cornea regeneration	[87]
	cholesteric gel matrix	proliferation of human dermal fibroblasts within 3D encapsulation and mechanobiologically induced development of myofibroblasts	dermal tissue transplants	[89]
	bundle gel of nematic-like helical fibers	enhanced growth of human endothelial cells in 3D entrapment	peripheral angiogenesis	[104]
	cholesteric-like gel film	oriented 2D growth and osteogenesis of human mesenchymal stem cells	bone regeneration	[105]
genetically and chemically modified filamentous bacteriophages	nematic substrate	2D directional growth of hippocampal neural progenitor cells, preosteoblasts, and fibroblasts	spinal cord defect repair	[91–92]
	nematic, cholesteric, and smectic templates	ostegenesis and biomineralization	bone regeneration	[93–94]
chitin nanowhiskers	nematic gel matrix	templating CaCO_3_ crystallization	bone regeneration	[106]
thermotropic aromatic–aliphatic copolyesters	nematic phase	biocompativity for MC3T3 E1 cell proliferation in vitro and for immune cell activity in vivo	subcutaneous implants	[108]
cholesterol oligo(L-lactic acid)	smectic E_h_-phase template	degradable topography for 2D growth of fibroblasts provided by spiral dislocations and multilayer erosion-based molecular delivery	connective tissue implants	[109]
synthetic amphiphilic molecules	lamellar gel string	3D encapsulation and aligned growth of human mesenchymal stem cells and HL-cardiomyocytes	cardiac regeneration	[36]
	gel phase of nematic fibers	3D growth and release of encapsulated myoblasts	muscle regeneration	[103]
cytocompatible liquid crystal elastomers	porous matrix in smectic-A phase	2D/3D oriented growth of neuroblasts, dermal fibroblasts, myoblasts, and skeletal muscle cells with tuned porosity and crosslinking density	nerve, skin, and muscle regeneration	[99–101]
	3D assembly of nematic microspheres	myoblast growth with controlled elasticity, porosity, and surface roughness	muscle regeneration	[102]

#### Dense collagen matrices and scaffolds in cholesteric organization

Various artificial systems have been developed by mimicking native LC organizations in living tissues [85]. For instance, Giraud–Guille and colleagues investigated hierarchically ordered surfaces of dense collagen in the cholesteric phase for the use of tissue regeneration templates for aligned cell growth with controlled functions [86]. The collagen fibrils, which are animal tissue-derived biopolymers used in pharmaceutics, cosmetics and food industries, have a strong advantage as a cell scaffold material in that they harbor specific amino acid sequences (such as Arg–Gly–Asp) on which cell attachment occurs through the binding of transmembrane receptors (such as integrin) [40]. Locally ordered collagen type-I gels, obtained by solvent evaporation, could induce an aligned 2D growth of human mesenchymal stem cells as well as their guided differentiation into bone tissues within two weeks in osteogenic media [86]. The concentrated (ca. 90 mg/mL) collagen type-I film exhibited a high stiffness (570 kPa of Young’s modulus). Optical transparency of the dense collagen film in the visible spectral range could be maintained after formation of an epithelium of human corneal epithelial cells in vitro [87]. The cornea-like 3D plywood cholesteric organization of the collagen matrix was confirmed by a nonlinear optics technique [88]. These properties enable the exploitation of the LC collagen matrix as a potential corneal implant. This matrix can be also utilized as a biological template for silica hybridization with long-range ordered structures [89]. Such 3D ordered architectures of hydrated dense collagen matrices allowed for the penetration of human dermal fibroblast from the surface into the matrix through the expression of metalloproteinases (collagenase I and gelatinase A), in which long-term migration and high-yield proliferation of cells were achieved, comparable to the cell density estimated in human connective tissues. Additionally, the mechanical feedback from the matrix to the cell body could stimulate the development of myofibroblast phenotype. This implicates that the LC matrix of dense collagen fibrils can be used as a biocompatible and biodegradable 3D scaffold for developing dermal equivalents.

#### Rod-like virus-based templates in nematic and cholesteric orders

Viral nanoparticles recently emerged as building blocks for self-assembled functional biomaterials [90]. The tissue engineering applications of viral LC architectures have been explored by Lee and colleagues [91–94]. Specifically, substrates of ordered rod-like viruses in the nematic phase have been utilized for directed 2D growth of cells. In this case, viruses are genetically modified to express integrin-binding motifs on their major coat proteins by using phage display techniques. Structurally ordered films have been fabricated by applying unidirectional shear force on a drop of concentrated M13 virus suspension, which resulted in a nematic-like organization of the viruses. The long-range order 2D nematic topography can guide the directional growth of hippocampal neural progenitor cells, preosteoblasts and fibroblasts, which has a potential for the treatment of spinal cord injuries [91–92]. Osteogenesis and biomineralization can be induced by using 2D substrate matrices made of M13 phages and M13-based peptide amphiphiles, which are in hierarchical nematic, cholesteric, and smectic-like nanoscale topographies [93–94].

#### LC elastomer scaffolds in nematic and smectic-A phases

Liquid crystal elastomers (LCEs) are crosslinked 3D networks of rubber-like synthetic polymers exhibiting orientational and/or positional order [95]. Due to the tunable malleability and highly anisotropic shape change in response to external stimuli, LCEs have been used as biologically relevant soft actuators, often being considered as artificial muscles [96], as first proposed by de Gennes [97]. However, LCEs exhibit a hydrophobic nature and lack cell adherence sites. Thus, in order to be applied for tissue engineering purposes, LCEs need to undergo additional physicochemical modifications. For example, Abbott and co-workers developed a cell-culture monitoring tool with which the orientational order of ECM-decorated nematic LCE substrates could be utilized for sensing ECM-attachment and proliferation of human embryonic stem cells [7,98]. Investigations by the Hegmann group have shown that bioengineered LCE scaffolds can be used to control mechanical response, growth direction, and proliferation rate of the seeded myoblasts and neuroblastomers. Porous LCE matrices in smectic-A with interdigitated cholesterol moieties induced 2D and 3D aligned cell growth in response to the lamellar molecular structure of LCE matrices, with tunable porosity and crosslinking density [99–101]. In addition, the 3D anisotropic cell scaffolds can be fabricated by using nematic LCE microspheres as building blocks by tuning elastic modulus, porosity, surface roughness, and local anisotropy [102].

#### LC hydrogels and implants in nematic and smectic-A phases

LC-ordered anisotropic gels have great potential to be directly applied to regenerative medicine therapeutics, due to their intrinsic tissue compatibility. Thermally or electrostatically induced phase transitions from isotropic sol to nematic gel of synthetic nanofibers or filamentous bacteriophages can be adopted as a mild and efficient process for cell encapsulation in a highly anisotropic hydrogel scaffold [91,103]. These in situ cell inclusion techniques allow for the production of physiologically functional tissue replicas of nerves by controlling 3D cell alignment and growth direction. When cardiomyocytes are embedded in the anisotropic gel scaffolds, the guided cell growth parallel to the major axis of the bundle structure enables the long-range propagation of action potential along the bundle gel. Collagen-based helical nanofibrillar scaffolds have shown the ability to support the growth of human endothelial cells [104]. The nanofibrils were generated by applying shear stress on a collagen solution in a (chiral) nematic phase. When the cells were seeded in a 3D hydrogel composed of the aligned nanofibrils, enhanced cellular outgrowth was induced via augmented expression of integrin α1, which resulted in arteriogenesis and blood perfusion recovery in the mouse ischemia model. In another study, hydrogel films made of concentrated collagen type I showed regular local alignments, which resulted in directed and oriented growth of human mesenchymal stem cells and their osteogenic differentiation [105]. Additionally, biomineralization-mimicking hybrid materials, using chitin nanowhisker gel matrices in a nematic phase as templates for CaCO_3_ crystallization, have been reported [106]. Lamellar phase PLA-*b*-PEG-*b*-PLA hydrogels can be made to exhibit better biodegradability and higher swelling behavior than isotropic gels for medical purposes [107]. Thermotropic copolyester materials in nematic order can be also synthesized to be used as a bioresorbable and tissue-compatible subcutaneous implant [108].

#### Synthetic substrates, membranes, and dispersions in other LC geometries

Smectic-like and columnar-like architectures can be also adopted as nanostructures for tissue engineering and regenerative medicine. Lamellar templates in the smectic phase with spiral dislocation domains have been fabricated by Stupp and co-workers, based on the self-assembly of cholesteryl oligo(L-lactic acid) [109]. In this system, the incorporation of the cholesteryl moiety and the short lactic acid chain enables cell membrane adherence and biodegradability, respectively. Thus, the layer-by-layer erosion can be utilized as a repeated delivery mechanism of drug molecules to the adhered cells on the interface. However, the biophysical interaction between the adhered cells and the nanotopography of the spiral dislocation structure remains to be revealed. Viscoelastic strings of lamellar peptide amphiphiles can be made to encapsulate living cells in an anisotropic monodomain gel [36]. The gel self-assembly is induced by cooling within a physiological temperature range via nozzle-protrusion into the salty buffer. During the process, human mesenchymal stem cells and HL-1 cardiomyocytes were shown to be entrapped and aligned along the string axis. When the cardiomyocytes were proliferated in the scaffold bundle, long-distance propagation of intercellular action potential could be achieved, demonstrating a functional role of the scaffold bundle in cardiac syncytium. As another application, lamellar lipid membranes can be used as a synthetic model system of *stratum corneum*, the outermost layer of epidermis, for evaluating the transdermal permeation efficiency of drug molecules in vitro [110], which has an implication in skin disease study [111]. Moreover, topical formulations of LC dispersions in various phases (e.g., lamellar, hexagonal, reverse hexagonal, bicontinuous cubic, micellar cubic) have been investigated in terms of the physicochemical interactions between *stratum corneum* and the LC formulations for pharmaceutic purposes [112–113].

## Conclusion

The strength of LC biomaterials lies in their self-assembly behavior and the formation of hierarchical 3D nanoarchitectures with long-range order. This spontaneous organization has strong advantages such as structural biomimicry, facile bottom-up processing, straightforward upscaling, and low energy cost. In tissue engineering applications, the self-assembly of biopolymers in biocompatible ranges of physicochemical factors can be an ideal property, especially for developing scaffolds for cell encapsulation. The local anisotropy of the 3D architecture in the LC scaffolds provides a versatile strategy to dictate guided cell growth and morphology control, which can be further extended to directed cell differentiation. Moreover, the tissue-mimectic characteristics of LC biomaterials can be exploited for fabricating artificial organs-on-a-chip and bioinspired drug delivery systems. The tunable optical response of biological LC materials is an additional benefit for inventing sensor devices for biomedical applications.

In spite of these advantages and functionalities, the application of LC nanoarchitectures in the field of tissue engineering still remains in its infancy, when compared to the advance in the applications to LC display electronics during the last decades. Technological breakthroughs can be achieved by introducing stimuli-responsivity or intelligence into the conventional LC biomaterials [114]. Hybridization with functional nanomaterials (e.g., stimuli-sensitive polymers, magnetic nanoparticles, carbon nanotubes and graphene-based materials) could widen practical uses in biomedicine. In addition, a combination with 3D printing techniques will open a new way to build complex 3D tissue constructs [115]. More detailed understanding on the structural change in LC scaffold induced by the degradation and remodeling by embedded cells and surrounding tissues is required. [116]. LC nanoarchitectures formed by hydrated lipids are also an important class that is to be investigated for tissue engineering purposes [117–127]. Biophysical roles of defect structures appearing in biological LCs [29,31–32] in cell–matrix interactions needs to be further explored for a more sophisticated synthesis of bioengineered tissue scaffold materials. Ultimately, the future research should be directed towards active LC scaffolds that can exhibit self-regulation and self-organization in dynamic coherence within local microenvironments in vivo, similar to our native tissue function [128].
